# Rapid spread of influenza A(H1N1)pdm09 viruses with a new set of specific mutations in the internal genes in the beginning of 2015/2016 epidemic season in Moscow and Saint Petersburg (Russian Federation)

**DOI:** 10.1111/irv.12389

**Published:** 2016-05-27

**Authors:** Andrey Komissarov, Artem Fadeev, Maria Sergeeva, Sergey Petrov, Kseniya Sintsova, Anna Egorova, Maria Pisareva, Zhanna Buzitskaya, Tamila Musaeva, Daria Danilenko, Nadezhda Konovalova, Polina Petrova, Kirill Stolyarov, Elizaveta Smorodintseva, Elena Burtseva, Kirill Krasnoslobodtsev, Elena Kirillova, Lyudmila Karpova, Mikhail Eropkin, Anna Sominina, Mikhail Grudinin

**Affiliations:** ^1^ Research Institute of InfluenzaMinistry of Healthcare of the Russian FederationLaboratory of Molecular Virology and Genetic EngineeringWHO‐recognized National Influenza Centre of Russian FederationSaint PetersburgRussia; ^2^ Research Institute of InfluenzaMinistry of Healthcare of the Russian FederationLaboratory of Influenza VaccinesSaint PetersburgRussia; ^3^ Research Institute of InfluenzaMinistry of Healthcare of the Russian FederationLaboratory of Molecular Virology and Genetic EngineeringSaint PetersburgRussia; ^4^ Research Institute of InfluenzaMinistry of Healthcare of the Russian FederationLaboratory of Evolutionary Variability of Influenza VirusesWHO‐recognized National Influenza Centre of Russian FederationSaint PetersburgRussia; ^5^ Research Institute of InfluenzaMinistry of Healthcare of the Russian FederationDepartment of ITWHO‐recognized National Influenza Centre of Russian FederationSaint PetersburgRussia; ^6^ Research Institute of InfluenzaMinistry of Healthcare of the Russian FederationLaboratory of BiotechnologyWHO‐recognized National Influenza Centre of Russian FederationSaint PetersburgRussia; ^7^ Federal Research Center of Epidemiology and Microbiology named after N. F. GamaleyaWHO‐recognized National Influenza Centre of Russian FederationMoscowRussia; ^8^ Research Institute of InfluenzaLaboratory of Influenza and ARI EpidemiologyWHO‐recognized National Influenza Centre of Russian FederationSaint PetersburgRussia

**Keywords:** A(H1N1)pdm09, high‐throughput nucleotide sequencing, influenza, Russia

## Abstract

A dramatic increase of influenza activity in Russia since week 3 of 2016 significantly differs from previous seasons in terms of the incidence of influenza and acute respiratory infection (ARI) and in number of lethal cases. We performed antigenic analysis of 108 and whole‐genome sequencing of 77 influenza A(H1N1)pdm09 viruses from Moscow and Saint Petersburg. Most of the viruses were antigenically related to the vaccine strain. Whole‐genome analysis revealed a composition of specific mutations in the internal genes (D2E and M83I in NEP, E125D in NS1, M105T in NP, Q208K in M1, and N204S in PA‐X) that probably emerged before the beginning of 2015/2016 epidemic season.

## Introduction

Influenza viruses are constantly evolving posing a significant threat for public health, therefore constant monitoring of genetic and antigenic properties is required for detection of newly emerging strains and annual revision of influenza vaccine composition. WHO‐recognized National Influenza Centers (NIC) in Moscow and Saint Petersburg collect and analyze information on the influenza activity in Russian Federation and perform antigenic and genetic analysis of circulating strains.

**Figure 1:**
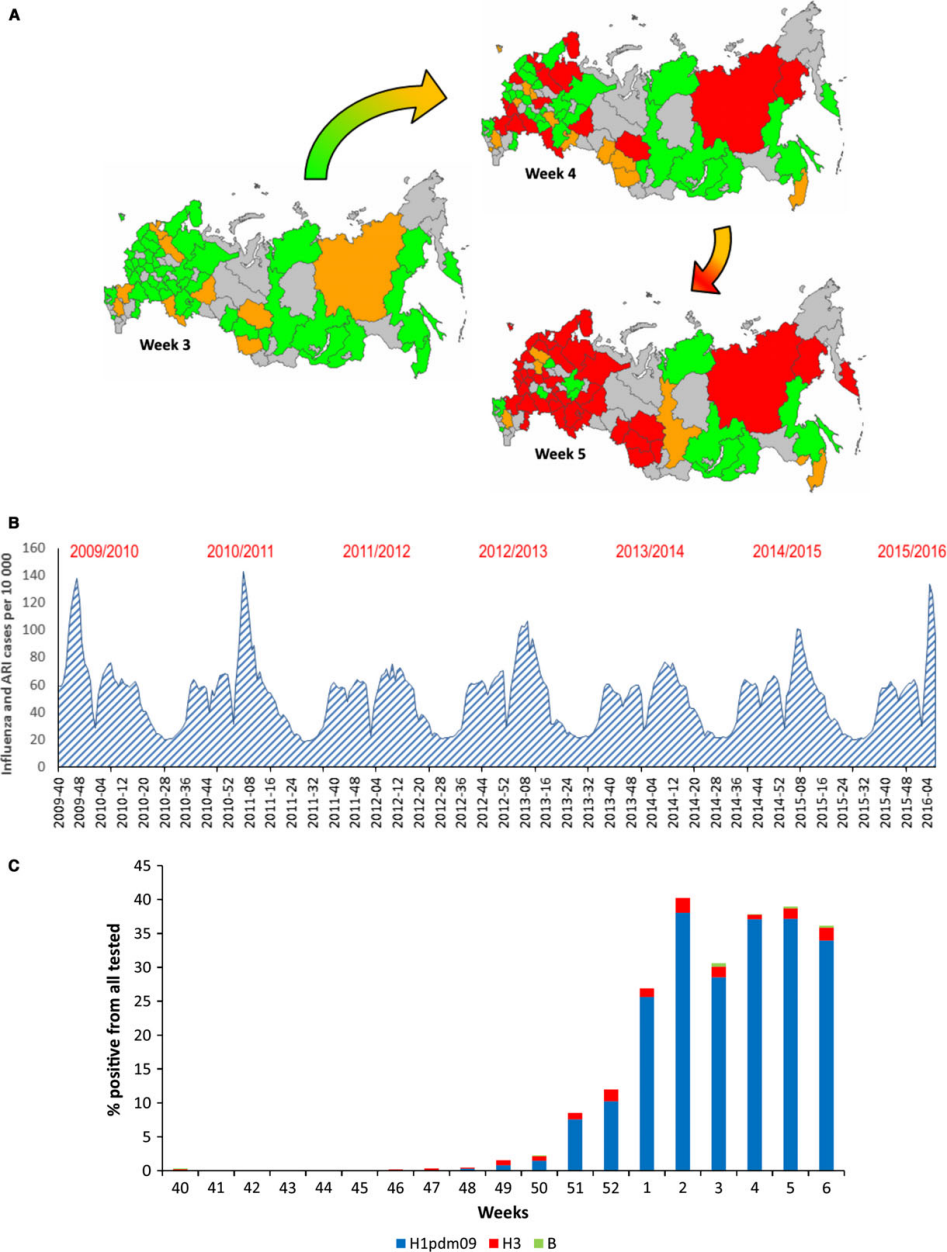
A. Influenza and ARI morbidity in Russian Federation on weeks 3–5, 2016. Epidemic threshold exceeded by less than 20% is indicated in green, epidemic threshold exceeded by 20 ‐ 50% is indicated by orange and epidemic threshold exceeded by more than 50% is indicated by red. B. Influenza and ARI incidence in Russian Federation in epidemic seasons 2009/2010, 2010/2011, 2011/2012, 2012/2013, 2013/2014, 2014/2015, and 2015/2016. C. Laboratory‐confirmed influenza cases in epidemic season 2015/2016, presented as % positive from all tested samples.

2015/2016 epidemic season in Russia is characterized by rapid increase of influenza and acute respiratory infection (ARI) morbidity with exceeding the epidemic thresholds in all federal districts in a short time period (Figure 1A).

An increase of influenza activity was registered from week 3 of 2016 (11‐17·01·2016). Increases in influenza‐like illness (ILI) and ARI incidence and hospitalization rates were registered up to week 5 when the epidemic peak was reached. These indicators were similar to those observed during the 2009 pandemic and epidemic in 2010‐2011 and were much higher than in other seasons (Figure 1B).

The number of cases with rRT‐PCR confirmed influenza A(H1N1)pdm09 increased from 28·5% in week 3 to 37·2% in week 5 of 2016. Influenza A(H3N2) and influenza B cases were detected sporadically (Figure 1C).

## Antigenic analysis of influenza A(H1N1)pdm09 viruses

Specimens used for virus isolation and sequencing were collected from 25·11·2015 (when the first PCR‐positive clinical samples were obtained) to 18·01·2016.

About 108 strains were characterized antigenically in both WHO NICs in Moscow and Saint Petersburg using WHO diagnostic kit and ferret and rat polyclonal antisera to the reference strains^1^. Most of the tested viruses reacted within two‐ to fourfold difference with antisera raised against the A/California/07/2009 virus.

Detailed antigenic analysis was performed for strains isolated in the NIC in Saint Petersburg (see Table 1). The hemagglutination inhibition (HI) test showed that all tested viruses were antigenically closely related to the vaccine strain as well as with the reference strains such as A/Hong Kong/5659/12 (cell‐derived) and A/Bolivia/559/13 (egg‐derived). They also reacted within two‐ to fourfold difference with antisera raised against contemporary reference influenza A(H1N1)pdm09 viruses isolated in Russia in 2009–2014. These results clearly demonstrate that most of the A(H1N1)pdm09 viruses isolated in the beginning of the epidemic season in Russia are antigenically A/California/07/2009‐like, for example, similar to the vaccine strain.

**Table 1:** Antigenic analysis of influenza А(H1N1)pdm09 viruses, isolated in Russia in the beginning of epidemic season 2015‐2016

Influenza viruses	Diagnostic antiserum (CDC) H1N1pdm (FR‐188)	Rat polyclonal antisera against influenza viruses							
		A/Cal/07/09	A/SPb/RII56/09	A/Cristchurch 16/10	A/SPb/RII27/11	А/НК/5659/12	A/SPb/RII26/13	A/SPb/RII06/14	А/Bol/559/13
A/California/07/2009	**2560**	**640**	320	160	320	160	640	320	320
A/St.Petersburg/RII56/2009	2560	640	**640**	160	160	160	320	320	320
A/Cristchurch/16/2010	1280	640	320	**1280**	160	80	160	320	320
A/St.Petersburg/RII27/2011	2560	640	640	320	**320**	160	320	640	320
А/НongКong/5659/2012	1280	320	320	320	320	**320**	640	640	320
A/St.Petersburg/RII26/2013	1280	320	320	160	320	160	**640**	640	320
A/St.Petersburg/RII06/2014	2560	320	320	320	320	320	640	**1280**	640
А/Bolivia/559/2013	2560	640	640	320	160	160	640	640	**640**
A/St.Petersburg/RII349/2015	1280	320	320	320	160	160	320	640	320
A/St.Petersburg/RII350/2015	1280	320	160	320	160	160	320	320	320
A/St.Petersburg/RII02/2016	1280	320	320	160	160	160	320	320	160
A/St.Petersburg/RII07/2016	1280	160	160	160	80	160	320	320	160
A/St.Petersburg/RII08/2016	1280	160	160	320	160	160	320	320	160
A/St.Petersburg/RII15/2016	1280	160	160	160	160	160	320	320	320

Homologous HI titers are shown in bold.

**Figure 2:**
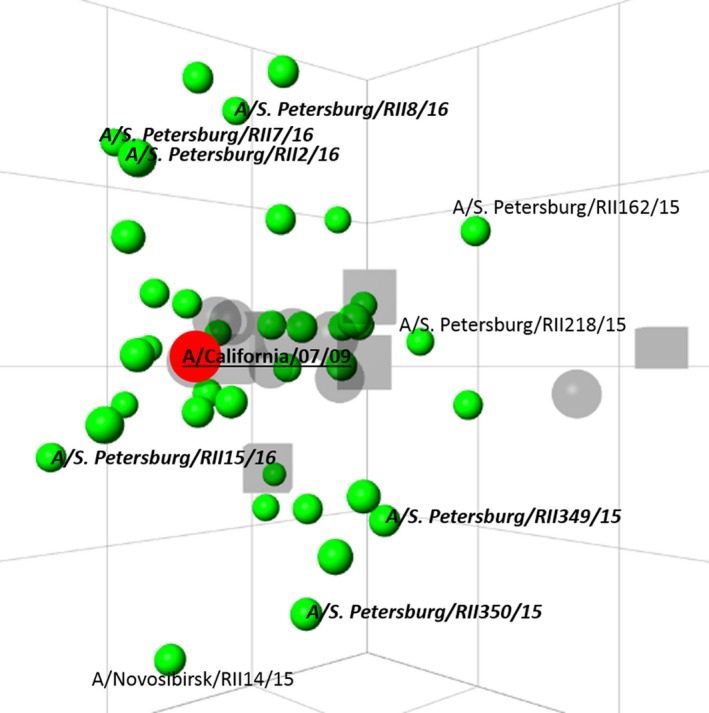
Antigenic map of influenza A(H1N1)pdm09 viruses isolated in Russia in 2009‐2016. The square on the map corresponds to a twofold difference in the homologous HI titer between the vaccine strain and the tested strain. Dots are viruses, squares—antisera. The vaccine strain is shown in red, with bold and underlined text. Strains isolated in epidemic season 2015/2016 are indicated in italicized bold.

Antigenic cartography was used for the analysis and visualization of the antigenic data^2^. The antigenic map (see Figure 2) based on the HI test demonstrates that the antigenic distance between the vaccine strain and the strains tested does not exceed the fourfold difference in homologous titer.

## Genetic characterization of influenza A(H1N1)pdm09 viruses

Full‐genome amplification of influenza A(H1N1)pdm09 viruses from 55 isolated strains (41 from Moscow, 13 from Saint Petersburg, 1 from Tomsk) and 22 clinical specimens (including 4 autopsy specimens) was performed according to Zhou *et al*.^3^ Nextera XT sample preparation was used to obtain libraries for next‐generation sequencing; full‐length genome sequences were obtained using Illumina MiSeq. All sequences were submitted to Epiflu GISAID database (isolates Acc. Nos. EPI_ISL_205809, 207503‐207574, 208228, 208230‐208232) (for detailed information, see Supplement 2).

**Figure 3:**
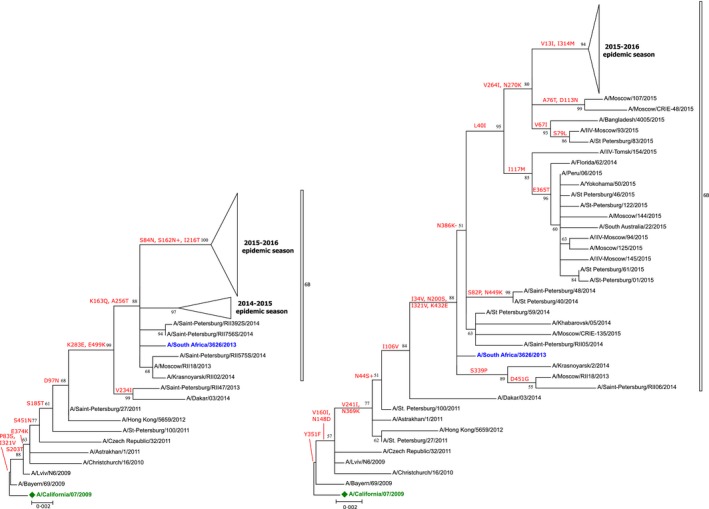
Maximum‐likelihood phylogenetic trees based on HA (left) and NA (right) nucleotide sequences of influenza A(H1N1)pdm09 viruses. Vaccine strain is labeled with a green diamond; clade representative strains are highlighted in blue. Amino acid substitutions are indicated above corresponding branches. Bootstrap analysis, 1000 replications. Similar strains are collapsed to triangles (for expanded tree, see Supplement 1). Phylogenetic analysis was conducted in MEGA6 [9].

According to the phylogenetic analysis of the hemagglutinin (HA) gene all viruses belong to 6B clade (A/South Africa/3626/2013‐like) clustering in a separate subclade (except A/IIV‐Tomsk/154/2015) bearing amino acid substitutions S84N, S162N, and I216T (Figure 3). Substitution S162N leads to gain of potential N‐glycosylation site. None of the substitutions were located in known antigenic sites. In some A(H1N1)pdm09 sequences from autopsy samples, substitution D222G was observed. Influenza A/IIV‐Tomsk/154/2015 virus clustered with the strains of 2014/2015 epidemic season (see Figure 3).

All viruses contained several substitutions in the neuraminidase of unknown functional significance (V264I, N270K, and N386K). N386K leads to loss of a potential N‐glycosylation site. Position 264 might be involved in viral host adaptation^4^.

Surface glycoprotein genes HA and NA are usual targets for molecular surveillance in WHO's Global Influenza Surveillance and Response System (GISRS) laboratories. Less attention is paid to evolutionary changes in the internal genes (PB2, PB1, PA, NP, M, and NS). We looked through all known influenza virus ORFs for amino acid substitutions. The distribution of identified substitutions among A(H1N1)pdm09 viruses circulating in the Southern and Northern Hemispheres in 2013, 2013/2014, 2014, 2014/2015, 2015, and 2015/2016 epidemic seasons was analyzed using EpiFlu GISAID database. Only full‐length genome sequences were included in the analysis: 2013—22 sequences, 2013/2014—188 sequences, 2014—28 sequences, 2014/2015—313 sequences, 2015—223 sequences, and 2015/2016—182 sequences).

## Set of specific mutations in internal genes

**Figure 4:**
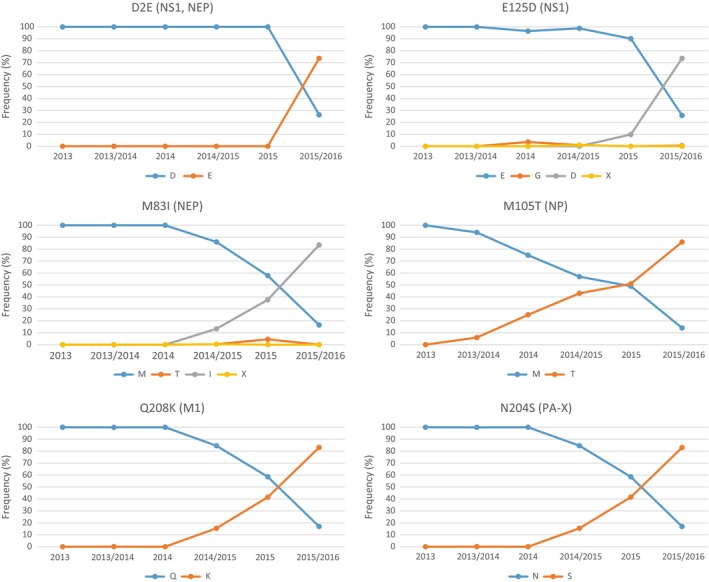
Frequency plots of selected amino acid substitutions in proteins encoded by internal genes of influenza A(H1N1)pdm09 viruses submitted to EpiFlu GISAID from 2013 to 2016.

Amino acid substitutions D2E, N48S, and E125D were identified in the NS1 protein. Substitutions D2E and E125D occurred in all viruses except for A/IIV‐Tomsk/154/2015 and N48S in 39% of sequenced viruses. An EpiFlu database search revealed that the frequency of substitutions D2E and E125D in NS1 protein of influenza A(H1N1)pdm09 viruses drastically increased in less than 1 year from 10% in 2015 in the Southern Hemisphere epidemic season to 74% in 2015/2016 in the Northern Hemisphere epidemic season. In the NEP protein, the same phenomenon was observed for the substitution M83I flanking nuclear export signal (NES), the frequency of which has increased from 13% in 2014/2015 epidemic season to 83% in 2015/2016 epidemic season. All strains from Saint Petersburg and Moscow bear this substitution (Figure 4).

Amino acid substitution M105T was found in the NP protein of all studied strains except for A/IIV‐Tomsk/154/2015. The M105T substitution frequency in EpiFlu GISAID database submitted influenza strains increased from 6% in 2013/2014 epidemic season to 86% in 2015/2016 epidemic season (Figure 4).

The amino acid substitution Q208K in M1 protein was predominant in the influenza A(H1N1)pdm09 viruses from Moscow and Saint Petersburg. First registered in the 2014/2015 epidemic season, the Q208K substitution reached 83% frequency in 2015/2016 epidemic season (Figure 4).

PA‐X is a small alternative product expressed from segment 3 of influenza A virus (IAV) genome. All sequenced strains encode substitution N204S in PA‐X. The frequency of this substitution increased during the past two epidemic seasons from 14·8% in 2014/2015 to 81·2% in 2015/2016 (Figure 4).

## Discussion

**Figure 5:**
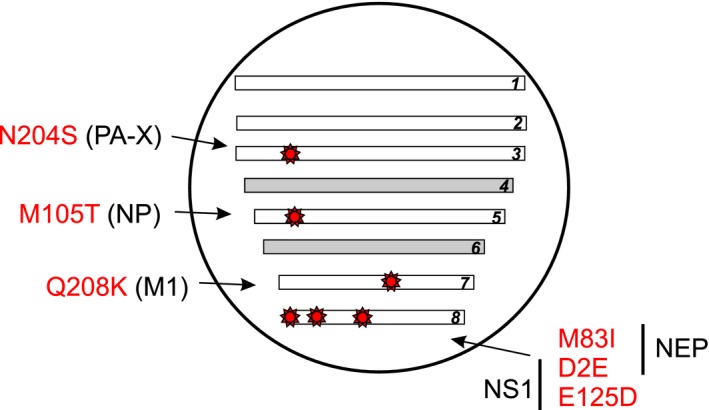
Specific mutations in the internal genes of influenza A(H1N1)pdm09 viruses dominating in the beginning of 2015/2016 epidemic season.

The beginning of 2015/2016 influenza epidemic season in Russia was marked by early and dramatic increase in ILI/ARI incidence in comparison with preceding epidemic seasons. Epidemiological indicators in week 3 of 2016 reached the levels observed during the peak of pandemic in 2009, when a new pandemic virus spread in a mainly naïve population. A significant number of lethal cases were registered in many regions of the Russian Federation. Genetic analysis of 77 influenza A(H1N1)pdm09 viruses circulating in Moscow and Saint Petersburg in the beginning of 2015/2016 epidemic season showed that all of them antigenically matched with the vaccine strain recommended by WHO. Amino acid substitutions found in HA (S84N and S162N (CHO+), I216T) and NA (V264I, N270K, and N386K (CHO‐)) do not lead to significant changes in antigenic properties in the HI assay. Detailed analysis of substitutions in the proteins encoded by internal genes showed that all viruses from Saint Petersburg and Moscow bear gene constellation, represented by specific amino acid changes: D2E and M83I in NEP, E125D in NS1, M105T in NP, Q208K in M1, and N204S in PA‐X (Figure 5). E125D in NS1 is known to be one of the key substitutions involved in shutdown of host mRNA transport, restoring inherent disability of A(H1N1)pdm09 virus to efficiently control human cell gene expression^5^. NS1 of all seasonal human influenza viruses (H1N1 seasonal and H3N2) contains D125 that interacts with cellular cleavage and polyadenylation factor 30 (CPSF30)^6^. Interaction with CPSF30 is absent in most animal‐adapted strains, so E125D substitution can be considered a milestone in host adaptation of influenza A(H1N1)pdm09 virus. Likewise NS1 protein, PA‐X is involved in host antiviral response shutoff^7^, ^8^, but the role of N204S substitution in PA‐X has not been studied before. Possible functional significance of other substitutions remains elusive.

It was a tempting idea to trace back this constellation of substitutions in internal genes to the strain in which it was detected for the first time. An EpiFlu GISAID database search showed that the first influenza strain bearing all the mentioned substitutions was A/New York/61/2015 isolated in August 2015. The evolutionary origin and stability of this constellation require further phylogenetic analysis. The observed rapid spread of influenza A(H1N1)pdm09 viruses with no significant antigenic changes in HA can be speculatively explained by increased transmissibility, as well as by increased virulence or by combination of both. The possible link between transmissibility or virulence and described internal gene constellation in influenza A(H1N1)pdm09 viruses awaits experimental proof.

## Addendum

All authors have contributed to, seen, and approved the manuscript. Andrey Komissarov and Artem Fadeev designed the study and wrote the manuscript. Andrey Komissarov, Artem Fadeev, Sergey Petrov, Maria Sergeeva, Kseniya Sintsova, Kirill Krasnoslobodtsev, and Anna Egorova performed the sequencing, genetic data analysis, and participated in the writing of the manuscript. Maria Pisareva, Zhanna Buzitskaya, and Tamila Musaeva performed rRT‐PCR testing and participated in the writing of the manuscript. Daria Danilenko, Polina Petrova, Elena Kirillova, and Nadezhda Konovalova performed virus isolation, HI assay, and antigenic cartography and participated in discussion. Lyudmila Karpova was responsible for epidemiological data acquisition; Kirill Stolyarov and Elizaveta Smorodintseva performed epidemiological analysis and data visualization and participated in the writing of the manuscript. Elena Burtseva, Anna Sominina, Mikhail Eropkin, and Mikhail Grudinin revised and approved the final manuscript.

## Supporting information


**Table S1** List of influenza strains submitted to EpiFlu GISAID database**.**

**Figure S1** Maximum‐likelihood phylogenetic trees based on HA (left) and NA (right) nucleotide sequences of influenza A(H1N1)pdm09 viruses**.**
