# Supplementary figures and images for: Rapid spread of influenza A(H1N1)pdm09 viruses with a new set of specific mutations in the internal genes in the beginning of 2015/2016 epidemic season in Moscow and Saint Petersburg (Russian Federation)
Source: Influenza Other Respir Viruses. 2016 May 27;10(4):247–53. doi: 10.1111/irv.12389 (PMC4910175; doi:10.1111/irv.12389)

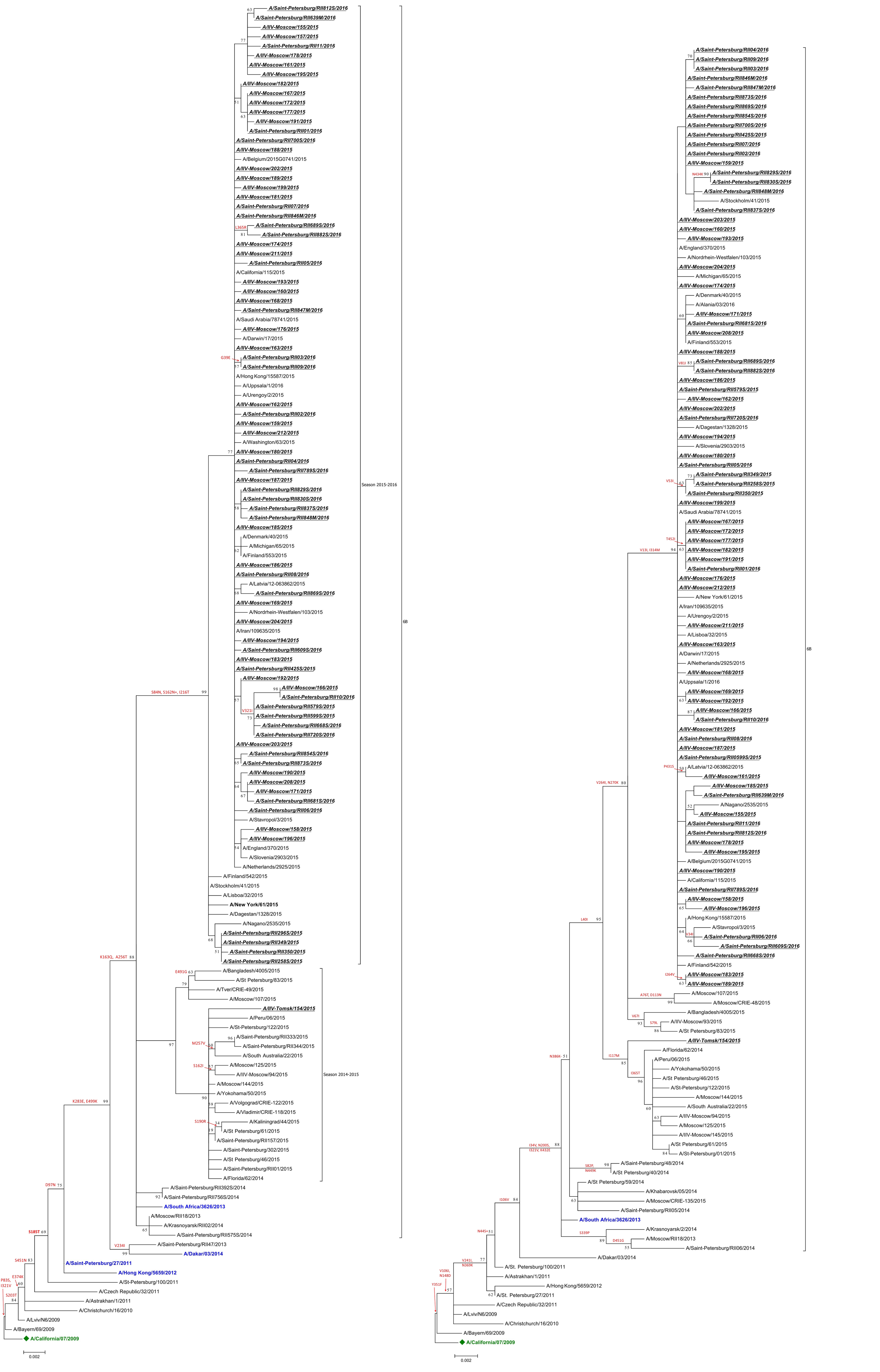

Supplement: Supplementary file 2 — Figure S1 Maximum‐likelihood phylogenetic trees based on HA (left) and NA (right) nucleotide sequences of influenza A(H1N1)pdm09 viruses. [file IRV-10-247-s002.tif]
